# Beyond choice architecture: advancing the science of changing behaviour at scale

**DOI:** 10.1186/s12889-021-11382-8

**Published:** 2021-08-10

**Authors:** Theresa M. Marteau, Paul C. Fletcher, Marcus R. Munafò, Gareth J. Hollands

**Affiliations:** 1 Department of Public Health and Primary Care, University of Cambridge, Cambridge, UK; 2 Department of Psychiatry, University of Cambridge, Cambridge, UK; 3 University of Bristol, School of Psychological Science, Bristol, UK; 4 Department of Public Health and Primary Care, University of Cambridge, Cambridge, UK

**Keywords:** Choice architecture, Behaviour, Changing behaviour

## Abstract

Addressing the global threats to population and planetary health requires changing many behaviours at scale. This demands consideration not only of the effect size of an intervention but also its reach – the proportion of the population exposed to the intervention.

We propose that a relatively under-researched and generally poorly specified set of interventions involving changes to physical micro-environments – often referred to as Choice Architecture - has the potential to make a significant contribution to meeting this urgent challenge.

Realising the potential of Choice Architecture interventions requires integration of basic *–* i.e. laboratory-based *–* and applied *–* i.e. field-based *–* research, generating interventions that can be delivered at scale alongside advancing theory. We illustrate this with examples to highlight the complementarity of laboratory and field studies informed by and in turn updating the results of evidence synthesis. The examples comprise two sets of interventions – changing the relative availability of products and changing their size - to reduce consumption of meat, energy from food and alcohol across populations.

## Background

Behaviours that pose major threats to human and planetary health include smoking, physical inactivity, use of fossil-fuelled transport and excessive consumption of alcohol, ruminant meat and ultra-processed foods [1, 2]. Many of these behaviours are now the norm – i.e. engaged in by a majority of people in many countries [2, 3]. We therefore need interventions that have the potential to reach whole populations in an equitable and cost-effective way. This requires a solution-oriented approach [4], with approaches to behaviour change situated within well-articulated theoretical frameworks [5] to better align theoretical and methodological rigour with pragmatic relevance.

There is no single approach to changing behaviour. Several taxonomies and typologies of a wide range of interventions and approaches have been developed over the last decade. These include the Behaviour Change Wheel [6] the Behaviour Change Technique Taxonomy [7] Intervention Mapping [8] and the Typology for Interventions in Proximal Physical Environments [9]. While they have some overlap, the first three aim for comprehensiveness. By contrast, the latter typology – which forms the basis for this article – is distinct in focusing exclusively on interventions that involve changing environments as a basis for changing behaviour at scale. It therefore excludes interventions that target cognitive and emotional predictors of behaviour including motivation and other beliefs and attitudes that form the basis of many individual-level interventions, but which do not readily lend themselves to interventions that can be delivered at scale.

**Fig. 1:**
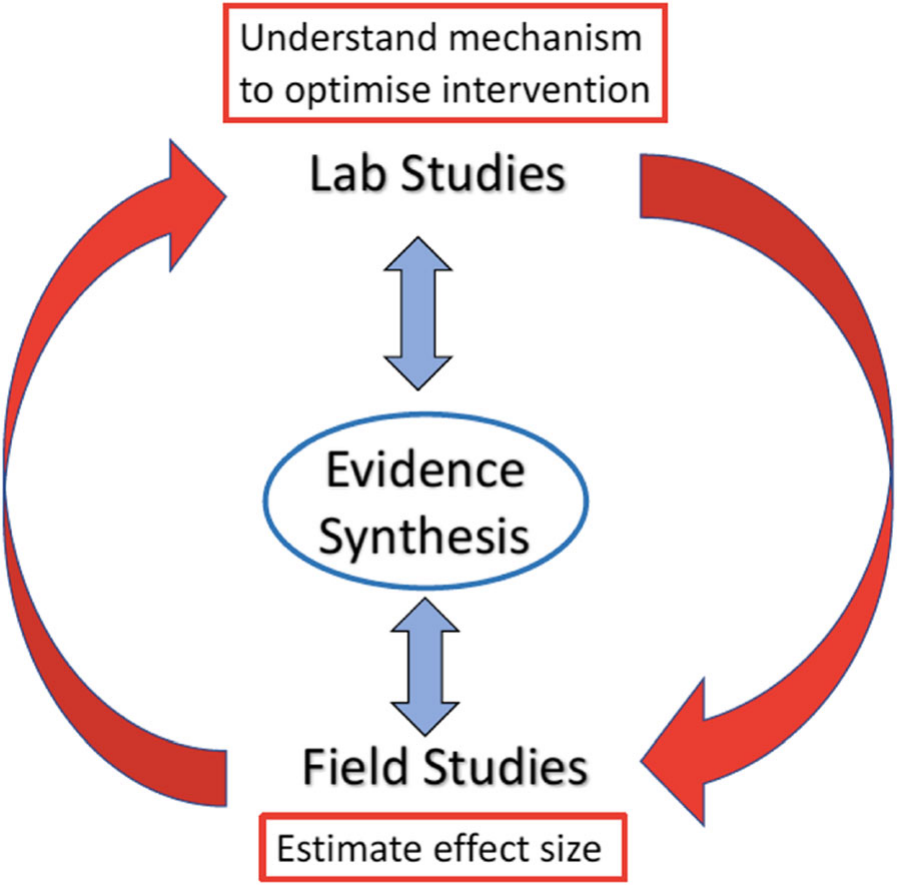
Building a robust evidence base using synergies between Evidence Synthesis, Laboratory and Field Studies

In keeping with a solution-oriented approach [4] our starting point is not from any one theory of behaviour change but rather from evidence of intervention effectiveness. Changing intentions has minimal impacts on behaviours that are routine or habitual [10]. In contrast, because such behaviours are primarily stimulus-driven, they can be significantly impacted by changing environments or settings [11, 12]. These effects are stronger than many people like to believe, as described by the fundamental attribution error or correspondence bias [13]. Although little researched, these effects are likely mainly realised through activating non-conscious processes [14, 15]. We acknowledge, however, that such a framework based on a dichotomy between conscience and non-conscious processes – while useful – is likely an oversimplification [16]. At their simplest, environments can be conceptualised as systems external to the individual comprising sets of related stimuli (Fig. 1) with individuals acting as agents that are dynamically responsive to structures and events in their environments, in effect forming an internalised model of that environment [17].

These environments vary in their type and can be physical, economic, social, commercial or digital. They also vary in scale which can be broadly divided into the micro level *–* i.e. settings with which and in which people interact and where the behaviour of interest occurs *–* and macro levels *–* i.e. higher-level sectors, systems and infrastructure that shape the micro level, such as health, education and economic systems, all levels of government and corporations and their governance [18]. These environments also overlap, and at any one time our behaviour is being shaped directly and indirectly by cues from one or more of these, often interacting. The precise nature of these environments and their interactions is currently poorly specified, reflecting the embryonic nature of systems approaches to understanding behaviour [19]. This does, however, provide a starting point for generating and understanding evidence of the effectiveness of intervening upon the environments that strongly shape our behaviour.

In considering the effectiveness of interventions for changing behaviour at scale, both effect size and reach – i.e. the magnitude of the effect of the intervention *and* the proportion of the population that are exposed to the intervention – are critical. Unlike interventions targeting individuals, population-level interventions may afford relatively small effects but still result in considerable population impact given the number of individuals they can reach. For example, standardised packaging of tobacco likely has very small effects at an individual level [20, 21] but at a population level these are important in contributing to reduced smoking rates [22, 23] with all the attendant health benefits. Two complementary approaches to both understanding and changing behaviour are discernible: those that focus on conscious volitional processes activated by desired goals or outcomes, and those that focus on non-conscious processes activated by environmental stimuli or cues. However, many of the theoretical frameworks used in psychological and behavioural science for changing behaviour are based on conscious, volitional processes. More comprehensive approaches, such as that embodied in the PRIME theory of human motivation [24], consider both intention and non-conscious processes. Nonetheless, a more comprehensive treatment of the latter is warranted given the uniquity of these processes in human behaviour and their relative neglect.

For example, all five of the theories used to develop a formal system for behaviour-change theories share these characteristics [25].

Given a focus on changing behaviour to improve population and planetary health, this is problematic for three reasons. First, many of the behaviours that need to change are routine or habitual, activated and sustained by environmental cues, so are least well explained by models using intentional processes [10]. While many interventions may be quick and simple such as action planning interventions [26] and those delivered using digital platforms [27], their requirement for a degree of cognitive engagement and attention necessarily constrain the numbers of people who will seek them out and engage with them. Second, interventions to change intentions or teach skills – while important for changing high risk individual behaviour – commonly cannot readily be scaled to reach whole populations. Third, such interventions can be more effective in those who are least socially deprived given their high demands on the cognitive, social and material resources of individuals that are unequally distributed across populations [28–30].

These problems can be illustrated using the example of obesity. There is no one cause of the global increase in BMI over the last 60 years. It has, however, occurred in parallel with changes in food environments, including increased availability of cheap ultra-processed packaged food served in growing portion sizes. These environments can act outside of awareness to cue consumption regardless of people’s intentions to do otherwise. Commercial weight loss programmes can achieve sustained weight loss, but this is not readily scaleable to reach whole populations. Finally, when interventions that target volitional processes to change diet are effective, they are more effective in those who are least socially deprived. By contrast, interventions that target environments are more equitable in their effects [31].

Our focus is upon one set of promising but under-researched interventions relating to cues that form part of physical micro-environments that have the potential to make a significant contribution to improving population and planetary health by changing behaviour at scale.

### Changing environments to change behaviour at scale: the example of physical micro-environments

These interventions have been described using a range of terms. In recent years, catalysed by the influential book *Nudge* [32], this has commonly included ‘Nudging’ and ‘Choice Architecture’. However, it is important to stress that these terms were originally developed within a general guiding framework that sets out underlying (philosophical) principles – libertarian paternalism – that can be applied to real-world problems. This framework was not intended to delineate the specific ways in which its principles can actually be applied to certain contexts, such as interventions to change health-related behaviour.

Inevitably, this means that the use of the terms has been nebulous and the original concepts obfuscated. The resulting lack of conceptual clarity when these terms have been applied to interventions to change behaviour, has led to a fragmented and uncertain evidence base. Achieving a consensus on terminology, or at least a recognition of the linked nature of evidence generated under different but conceptually related terms, has the potential to advance development of a coherent evidence base about what could work. This would strengthen the contribution that this approach can make to changing behaviour at scale. In other words, while it may not be possible to agree a single term and definition, we should at least recognise the overlap. Furthermore, when we use a concise shorthand expression such as *Choice Architecture* – a term that has general currency but lacks specificity – we should be clear what it represents rather than assuming a shared understanding.

While there have been attempts to map the core characteristics of Choice Architecture interventions, these have tended to focus on broad theoretical principles rather than pragmatic relevance [9, 12, 33]. To advance the generation and synthesis of evidence about interventions to change behaviour at scale, we also need to be able to describe their characteristics with greater clarity and precision, in this case the specific ways in which physical micro-environments are altered.

The TIPPME intervention typology (Typology of Interventions in Proximal Physical Micro-Environments) [9] attempts this by outlining six intervention types or ways to alter either the properties, or the placement, of objects or stimuli within proximal – i.e. sensorily perceptible – physical micro-environments, as applied to behaviours linked to food, alcohol and tobacco consumption. Placement can be manipulated in terms of whether a given object is present (Availability) and where it is located within an environment (Position). In turn, the properties of objects present within a given environment can be manipulated in respect to their Functionality, Presentation, Size and the Information available about them.

### Advancing the science of changing behaviour at scale

A more precise and pragmatically focused conceptualisation of the intervention of interest, set within well-articulated theoretical frameworks, is important. Choice Architecture is not a formal theory or model of behaviour. It can be understood as a form of *situationism* – a perspective built on observations that external environmental factors are often more predictive of behaviour than personality traits or motivation [11]. It is informed by ideas from reinforcement learning theories and machine learning that are central to neuroscience models of learning and decision making. These include distinguishing between model-free and model-based behaviour which could provide a basis for understanding mechanisms by which Choice Architecture interventions have their effects [12]. The theoretical frameworks for Choice Architecture interventions will evolve, informed by evidence of the effects of such interventions [4]. Thus, the development of theoretical frameworks for Choice Architecture interventions – and indeed other interventions to change behaviour – is only one part of building a robust evidence base. Even with that foundation in place it is too easy to overestimate what we know by extrapolating from a few preliminary studies or by assuming that a successful intervention will generalize from the laboratory to the real-world or from one real-world setting to another.

To avoid these errors, a robust evidence-base of behaviour change interventions require*s: i.* a summary of existing studies, preferably synthesised using rigorous systematic methods (e.g. Cochrane systematic reviews); *ii.* laboratory studies to understand mechanism and optimise interventions*; iii.* Replicated field studies conducted in real-world environments to estimate effect sizes. (Fig. 1).

We use the terms laboratory and field studies to distinguish between studies conducted in created and real-world settings, the former providing more experimental control than the latter but less ecological validity. We note, however, that this distinction is not always clear cut given that naturalistic laboratory restaurants, stores and bars are used to study eating, shopping and drinking, respectively [12].

In building a robust evidence base we prioritise three key elements - included in other approaches [34–36] – in which we place evidence synthesis at the centre, regularly updated with evidence from field and laboratory studies.

Evidence for an intervention’s effects on behaviour can be generated in a range of ways and sequences: there is no one optimal path to developing an evidence base for any given intervention. An evidence base can meaningfully start with a field study opportunistically arising from a policy change in the absence of evidence for its effectiveness. Evidence generation may also start with a laboratory study based perhaps on pre-existing indirectly relevant evidence. Both starting points are described below.

Using examples of two types of intervention – Availability and Size – we describe below the use of evidence synthesis, laboratory and field studies to illustrate the processes of building an evidence-base using synergies between these types of studies as depicted in Fig. 1. These two types of intervention are selected from the six types included in TIPPME because to date they have been the focus of more research with initial results suggesting they are likely the most promising. The potential for such interventions is illustrated in a recent review comparing the effect in field settings of three sets of interventions for healthier eating, categorised by the authors as mainly targeting cognitions, attitudes or behaviour. The latter category – which included interventions to reduce the size and availability of unhealthier products – generated the largest effect sizes in reducing unhealthier eating [37].

### Availability

Two systematic reviews highlighted an extremely limited evidence base for the effect of changing the availability of a food or drink upon the likelihood of its selection or consumption [38, 39]. They also revealed a lack of consistent conceptualisation for the intervention, which informed a conceptual framework [40]. This draws a distinction between interventions that alter the absolute number of options and those that keep these constant but alter their proportions. One field study provided evidence that increasing the proportion of lower energy food offered in a cafeteria reduces the energy (calories) purchased by about 7%. This effect has recently been replicated in a larger field study [41, 42]. Another field study provided evidence that doubling the proportion of plant-based meals (and correspondingly halving the proportion of meat-based meals) increased sales of plant-based meals by between 41 and 79% [43]. Laboratory studies complement these field studies to investigate the mechanisms by which interventions that change availability as a basis for optimising these [44, 45] and the contexts under which their effects may be greatest [46].

This evidence informs recent reviews [47] and will inform updates of pre-existing reviews [39, 48].

### Size

Of 72 studies included in a Cochrane review of size-based interventions on selection and consumption of food, tobacco and alcohol, 69 were food-related, three concerned tobacco and none concerned alcohol [48]. Across several meta-analyses, larger portions, packages and tableware resulted in people consistently eating more. A laboratory study assessing how wine glass size affects judgements of wine volume in ways that might affect consumption [49, 50] informed a series of field studies assessing the impact of wine glass size upon volume of wine sales [51–53]. These results from five bars and restaurants were synthesised in a mega-analysis [54]. This found no evidence of an effect of wine glass size on sales in bars, but a reduction of around 7% in restaurants. Two laboratory studies tested mechanisms by which the size of wine glasses might affect sales in bars and restaurants [45, 55]. The former found no evidence for any of the hypothesised mechanisms [55], a finding vindicated by the lack of an effect in field studies. The latter found evidence to support the hypothesised mechanism for an effect of wine glass size in restaurants, namely that when free-pouring from a wine bottle people pour more into larger capacity wine glasses. As found for food [48], when presented with larger quantities, people consume more.

This evidence has informed recent reviews [50] and will inform updates of the review that stimulated this programme of research.

Interventions should not be considered in isolation. A systems approach to changing behaviour at scale [19] predicts that synergies between interventions are possible, both within and across behavioural domains. For example, reducing the number of tobacco retailers in the context of an anti-tobacco mass media campaign may have a greater impact than either intervention implemented on its own [56–59]. Similarly, given that alcohol contributes about 8% of the energy intake of those consuming it, reducing alcohol consumption through any effective intervention would also help tackle obesity [60].

## Conclusion

International collaboration and co-ordination is needed across the many initiatives aiming to strengthen the science of behaviour change [61], the Human Behaviour Change Project [62] and the Science of Behavior Change programme [63]. These will need to include an explicit focus upon interventions that change behaviour at scale in order to make a significant contribution to the urgent global challenge to improve population and planetary health.

## Data Availability

Not applicable given this manuscript comprises an analysis of published studies.
